# Characterization of whitening toothpastes and their effect on the physical properties of bulk-fill composites

**DOI:** 10.1590/1678-7757-2022-0428

**Published:** 2023-05-15

**Authors:** GARCIA Raíssa Manoel, Waldemir Francisco VIEIRA, Danielle Ferreira SOBRAL-SOUZA, Flávio Henrique Baggio AGUIAR, Débora Alves Nunes Leite LIMA

**Affiliations:** 1 Universidade Estadual de Campinas Faculdade de Odontologia de Piracicaba Departamento de Odontologia Restauradora Piracicaba SP Brasil Universidade Estadual de Campinas, Faculdade de Odontologia de Piracicaba, Departamento de Odontologia Restauradora, Piracicaba, SP, Brasil.; 2 Instituto de Pesquisas São Leopoldo Mandic Campinas SP Brasil Instituto de Pesquisas São Leopoldo Mandic, Campinas, SP, Brasil.

**Keywords:** Toothbrushing, Coffee, Color, Activated charcoal, Operative dentistry

## Abstract

**Objective:**

To characterize activated charcoal and 2% hydrogen peroxide-based toothpastes and investigate their effects on roughness, color change, and gloss of bulk-fill composite resin.

**Methodology:**

Composite resin specimens (Aura Bulk Fill, SDI) were subjected to 5,000 brushing cycles with regular toothpaste (Colgate Total 12, [RT]), activated charcoal toothpaste (Bianco Dental Carbon, [AC]), or hydrogen peroxide-containing toothpaste (Colgate Luminous White Advanced, [HP]), with or without coffee exposure. The pH, particle characterization by scanning electron microscopy (SEM), and weight% of solid particles in the toothpaste were assessed. Roughness (Ra) was evaluated using a surface profile-measuring device, color change (∆E_ab_/∆E_00_) by reflectance spectrophotometer, and gloss unit (GU) by glossmeter. Kruskal–Wallis, Dunn, Friedman, and Nemenyi tests were used, and the correlation coefficient test was performed between Ra and GU (α=0.05).

**Results:**

RT presented a higher Ra after brushing and did not change after staining with coffee; moreover, the ∆E_ab_/∆E_00_ values were higher for RT than HP. Regarding gloss, AC and HP obtained higher values compared to RT. A significant negative correlation between gloss and Ra was found for RT exposed to coffee. All toothpastes had a neutral pH; however, RT had the largest percentage of solids by weight. SEM images showed particles of many sizes: irregular shape (RT), more regular particles (AC), and spherical clusters (HP). Although surface roughness, color change, and gloss may compromise the longevity of restorations, the whitening toothpastes tested did not promote more morphology changes than regular toothpastes.

## Introduction

Whitening toothpastes are low-cost over-the-counter products often used without professional advice from a dentist.^1^ The mechanism of action of these products is the removal of extrinsic staining by their abrasive ingredients (e.g., hydrated silica, calcium carbonate, alumina, pearlite), incorporating optical modifiers (e.g., blue covarine) and chemical activators (e.g., hydrogen peroxide and pyrophosphates).^2^ Other promising examples of whitening toothpaste are those containing activated charcoal^3^ and marketed by manufacturers with promises of potential detoxifying benefits, antiseptic and antifungal action, or as organic alternatives to conventional toothpastes.^3,4^ However, no clinical evidence suggests these benefits in the oral environment or the properties of the composites.

Composite resins have become the material of choice for restorations due to their ability to reproduce the shape, function, and beauty of natural-looking teeth.^5^ The use of bulk-fill composites in clinical practice is an attractive proposition since single increments of 4–5 mm thickness can be cured by light, resulting in a consequential reduction in clinical time.^6-7^ The basic aesthetic and surface characteristics of these composites, mostly color alteration, gloss, and roughness, are important factors that can affect the longevity and replacement of the restorations.^8^ The decrease in composite properties may be associated with adverse conditions existing in the oral cavity, such as attritional wear promoted by masticatory forces, toothbrushing, and changes in temperature and pH of the oral environment.^9-11^

When brushing is associated with whitening toothpastes, the surface of the restorations can be damaged due to the concentration of abrasive particles or other chemical agents that can change surface properties.^2,4,9-12^ This may lead to an increased surface porosity and removal of charged particles that induce water absorption.^9^ These alterations promote a change in surface smoothness, loss of gloss, and color change because the surface roughness also interferes directly with the intensity and rate of pigmentation.^13-14^ This is why it is imperative to assess the characteristics of these toothpastes and their effect on restorative materials.

Color stability is a crucial property of restorative materials, and color can be modified by intrinsic and extrinsic discolorations.^5,13^ Intrinsic factors are associated with material degradation due to components such as organic matrix composition and the initiator system, whereas the size, content, and hardness of the filler particles are directly influenced by photopolymerization.^14-15^ Extrinsic factors are associated with pigment adsorption and absorption derived from sources such as nicotine, medications, and the patient’s diet.^14-15^ Color stability can be compromised by chromogenic beverages, depending on the pH, and the frequency and duration of contact of these beverages with the material.^14^ Some common beverages consumed by the population have a greater potential to affect the color stability of composite resins, such as coffee, tea, juices, and red wine.^5^

The oral cavity is exposed to several factors daily, with the potential to alter the surface of restorations and cause aesthetic degradation.^14^ Thus, this study was proposed to evaluate the effect of whitening toothpastes containing hydrogen peroxide or activated carbon on the surface of bulk-fill composite resins, using a coffee staining protocol and simulated brushing. Two null hypotheses were tested: 1) charcoal-based and hydrogen peroxide-based toothpaste would not cause higher surface roughness than regular toothpaste; and 2) charcoal-based and hydrogen peroxide-based toothpaste would not cause higher color change and lower gloss than regular toothpaste.

## Methodology

### Experimental design

Two independent variables were evaluated: type of toothpaste and coffee exposure (staining). The dependent variables were roughness, color change, and gloss. Sample size consisted of 20 specimens in each group, totaling 120 specimens that provided 80% power (β=0.20) for minimal detectable effect size of 0.41 for toothpaste, 0.93 for staining, and 0.27 in the time factor, with a 5% significance level (α=0.05). Estimations were performed using the Gpower program.^16-18^ The physical characteristics of the toothpastes were evaluated by analyzing pH, particle characterization, and percentage weight of the solid particles. Additionally, scanning electron microscopy (SEM) was performed for qualitative analysis of the resin surface.

### Sample preparation

In total, 120 cylindrical specimens (universal shade) of composite resin (Aura Bulk Fill, SDI, Bayswater Victoria, Australia) were prepared in a silicon matrix (8.0 mm diameter, 4.0 mm thickness; Elite HD + normal setting Zermack). The increment of bulk-fill was placed in the silicon matrix and covered with a polyester strip (120×10×0.5 mm – Maquira, Maringá, PR) and glass slide, under a 500 g weight for 30 s.

The samples were light-cured with a light-emitting diode (Bluephase C8^®^ Light Unit Vivadent, Schaan, Lichtenstein) for 20 s in high-intensity mode, with 1120 mW/cm^2^ irradiance previously estimated by measuring the power of the light source with a power meter (Ophir Optronics Laser Measurement), according to the manufacturer’s instructions. The distance between the light source and the composite was 1 mm, representing the thickness of the glass slide. Next, the samples were removed from the mold, and the excess resin was removed with a #15 scalpel blade.

All samples were stored for 24 h at 37°C and 100% relative humidity before being subjected to the process using a polishing machine (Aropol 2V, Arotec, Cotia, SP, Brazil). The top surface of each sample was planned and polished for 1 min with 2500- to 4000-grit paper, using a water-cooled polishing machine followed by polishing cloths (Top, Ram e Supra, Arotec), and 1.0 and 0.25 µm diamond spray (Buehler, Lake Bluff, IL, USA). Afterwards, the samples were immersed in deionized water in an ultrasonic machine (Marconi, Piracicaba, SP, Brazil; Ultra Clearer USC-1450 A/Frequency 25 kHz; Unique) for 15 min to remove the residue left from the polishing processes.

**Figure 1:**
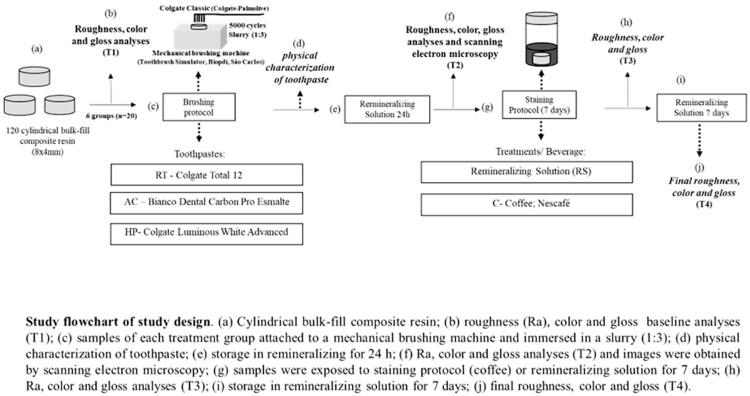
Study flowchar

**Figure 2:**
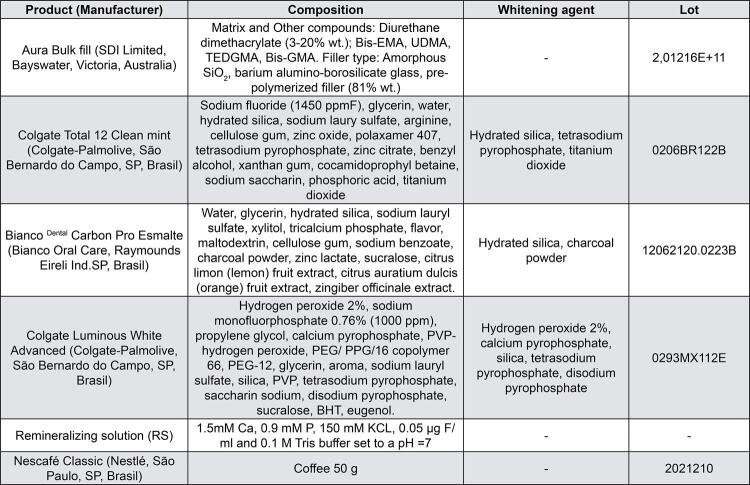
Description of products, including manufacturers, composition, lot, and pH

Abbreviations: Bis-EMA, bisphenol-A hexaethoxylated dimethacrylate; UDMA, urethane dimethacrylate; TEDGMA, triethylene glycol dimethacrylate; Bis-GMA, bisphenol-A diglycidyl ether dimethacrylate; * according to the manufacturers' recommendation.

The samples were randomly allocated into six groups (n=20) for subsequent treatments, as described in the study flowchart (Figure 1), according to the Ra baseline values. The color coordinate measurements and the gloss and roughness analyses are reported in greater detail below, and the product descriptions, including manufacturers and composition, are described in Figure 2.

### Characterization of the toothpastes

#### pH analysis of the slurries

The pH of the tested toothpaste slurries was analyzed in triplicate using a pH meter (Procyon, São Paulo, Brazil) before toothbrushing.^19^

#### Analysis of the percentage by weight of solid particles

A 40 mL sample of each slurry was weighed and then centrifuged (20 min, 3000 RFC, 25°C). The supernatant was removed carefully, and the residual solids were kept at 40°C for 10 days. Then, the dehydrated solid particles were weighed, and the weight percentages of solid particles were estimated regarding the total weight of the toothpaste slurry.^20^

Percentage weight of solid particles (%): [(P3–P2)/P1]×100

P1: Initial weight of toothpaste; P2: weight of Falcon tube; P3: final weight of toothpaste dehydrated with the Falcon tube.

#### Characterization of particles

The filler particle characterization of the toothpaste was adapted from a previous study.^21^ Approximately 1 g of each toothpaste was extruded onto a carbon tape, dried in an incubator at 37°C for 20 min, sputter-coated with gold and palladium particles, and observed under a scanning electron microscope (Jeol, JSM 5600LV, Tokyo, Japan), operated at 15 kV and 2000× magnification.

## Analysis of bulk-fill physical properties

Analysis of the physical properties (surface roughness – Ra, color change – CC, and gloss unit – GU) was performed at four time points: at baseline (T1), after 24 h of exposure to toothpaste (T2), after the staining protocol (T3), and seven days after immerging the samples in remineralizing solution (T4).

### Surface roughness (Ra)

Ra was performed using a surface profile measuring device (Mitutoyo Surfitest SJ-410, São Paulo, SP, Brazil). The readings were made in three different positions for each sample to obtain the mean roughness. The positions were altered 120° after each measurement to promote a homogeneous reading of the entire sample. The analysis was performed under the following parameters: 0.25 mm cut-off, 5 N static load, 3 mm run-up distance, and 0.5 mm/s speed.^22^

### Color

Color evaluation was performed using a reflectance spectrophotometer (CM 700D, Minolta, Osaka, Japan). The samples were placed on a Teflon device (sample holder) inside a light cabin (GTI MiniMatcher MM1e; GTI Graphic Technology Newburg, NY, USA) to standardize the measurement. Color change was estimated by the CIELAB system, which uses the following L*(luminosity), a*(green-red axis), and b*(blue-yellow axis) values: $\Delta E_{ab}={\left[{\left(\Delta L^{*}\right)}^{2}+{\left(\Delta a^{*}\right)}^{2}+{\left(\Delta b^{*}\right)}^{2}\right]}^{1/2}$ , and also by the CIEDE2000 system, which uses the following H (hue) and C (chroma) values: $\Delta E_{00}=[{\left(\Delta L^{\prime }/K_{L}S_{L}\right)}^{2}+(\Delta C^{\prime }/{K_{C}S_{C})}^{2}+{\left(\Delta H^{\prime }/K_{H}S_{H}\right)}^{2}+R_{T}\left(\Delta C^{\prime }/K_{C}S_{C}\right)*\left(\Delta H^{\prime }/K_{H}S_{H}\right)]\cdot .^{1/2}$ .^5,23^

### Gloss unit (GU)

GU value was measured using a glossmeter (Novo-Curve, Rhopoint Instruments, Hastings, UK). The glossmeter was calibrated before each use using a traceable calibration tile (Rhopoint Instruments) with low and high reference reflectivity. The reference on the glossy side of the tile measured 93.3 GUs at a 60° angle (ISO-Standards, ISSO 2813). Three measurements were made, corresponding to each quadrant of the sample. The mean reading was recorded as a unit of gloss.^24^

## Brushing protocol

The samples were attached to a mechanical brushing machine (Toothbrush Simulator, Biopdi, São Carlos, SP, Brazil), which used a 5 Hz frequency and a 200 g load^25^ and held the polished surface positioned upwards. All the samples were brushed with a soft toothbrush (Colgate Classic, Colgate-Palmolive, São Bernardo do Campo, SP, Brazil) positioned so that the toothbrush head was parallel to the sample. The samples were immersed in a slurry prepared with the tested toothpastes and distilled water in a 1:3 ratio by weight.^25^ Toothbrushing was simulated using 5,000 brushing cycles, corresponding to six months of brushing.^24,26^ Afterward, the samples were washed in distilled water, dried with paper towels, and stored in remineralizing solution (RS) (Table1) for 24 h before beginning the staining protocol. The RS was renewed every day during the experiment. Although remineralization actions are not expected in the composite resin, the purpose of using RS was to simulate the oral environment.^27^

**Table 1:** Characterization of the toothpaste (pH and % weight of solid particles)

Toothpaste	pH∞	% Weight of solid particles
Colgate Total 12	7.65	57.94
Bianco Dental Carbon Pro	7.12	54
Colgate Luminous White Advanced	8.25	42

∞ pH of the slurry - Analysis performed in triplicate with a pH meter (Procyon, São Paulo, Brazil) calibrated with standard values (pH 4.0 and 7.0).

A mark was made with a diamond drill on the edge of each sample to secure correct positioning of the sample on the mechanical brushing machine, thus ensuring its readability in the same direction (perpendicular to brushing). This mark also guided the analyses.

## Staining protocol

The surfaces (bottom and lateral) of the samples subjected to coffee staining were covered with sticky wax (Asfer, São Caetano do Sul, SP, Brazil) applied with an electric dripper (Plaster, Caxias do Sul, RS, Brazil), so that only the top surface was exposed to coffee.^27^ The samples were stained with a coffee solution for seven days and renewed daily at a standardized time.^27,28^ The solution was made by dissolving 3.6 g of coffee into 300 ml of boiled distilled water, following the manufacturer’s recommendations. The resulting solution was allowed to cool for 10 min at room temperature before use.^27^ The samples were stored in 3 mL of coffee, in a 7 mL acrylic device closed tightly to prevent changes in the coffee volume, and at 372°C for 24 h to stimulate the oral temperature. The samples were washed with distilled water at each replacement, and then dried with absorbent paper. After seven days, the samples were stored in remineralizing solution changed daily for more seven subsequent days to achieve color stabilization. The protocol lasted 14 days.

The samples receiving only brushing treatment were stored in remineralizing solution, changed daily for 14 days after the brushing cycles, in 100% relative humidity, and at a 372°C temperature. The pH (pH=4.90) of the coffee was evaluated in triplicate using a pH meter (Procyon, São Paulo, Brazil).

## Scanning electron microscopy

Four additional samples were examined for qualitative sample surface evaluation after exposure to the toothpastes. The samples were sputter-coated (Bal-Tex SCD 050 sputter coater, Germany) with gold and palladium particles and observed under SEM (Jeol, JSM 5600LV, Tokyo, Japan) operated at 15 kV and 2000× magnification.

## Statistical analysis

The descriptive and exploratory analyses were performed using R software. The data were tested for normal distribution (Shapiro-Wilk test) and equality of variance (Levene’s test), followed by a nonparametric test. Kruskal-Wallis and Dunn tests were performed for comparisons among the toothpastes, the Mann-Whitney test was used for comparisons with and without staining, and the Friedman and Nemenyi tests were used for comparisons between the time points. All analyses were performed considering α=0.05.

## Results

### Characterization of the toothpastes

**Figure 3:**
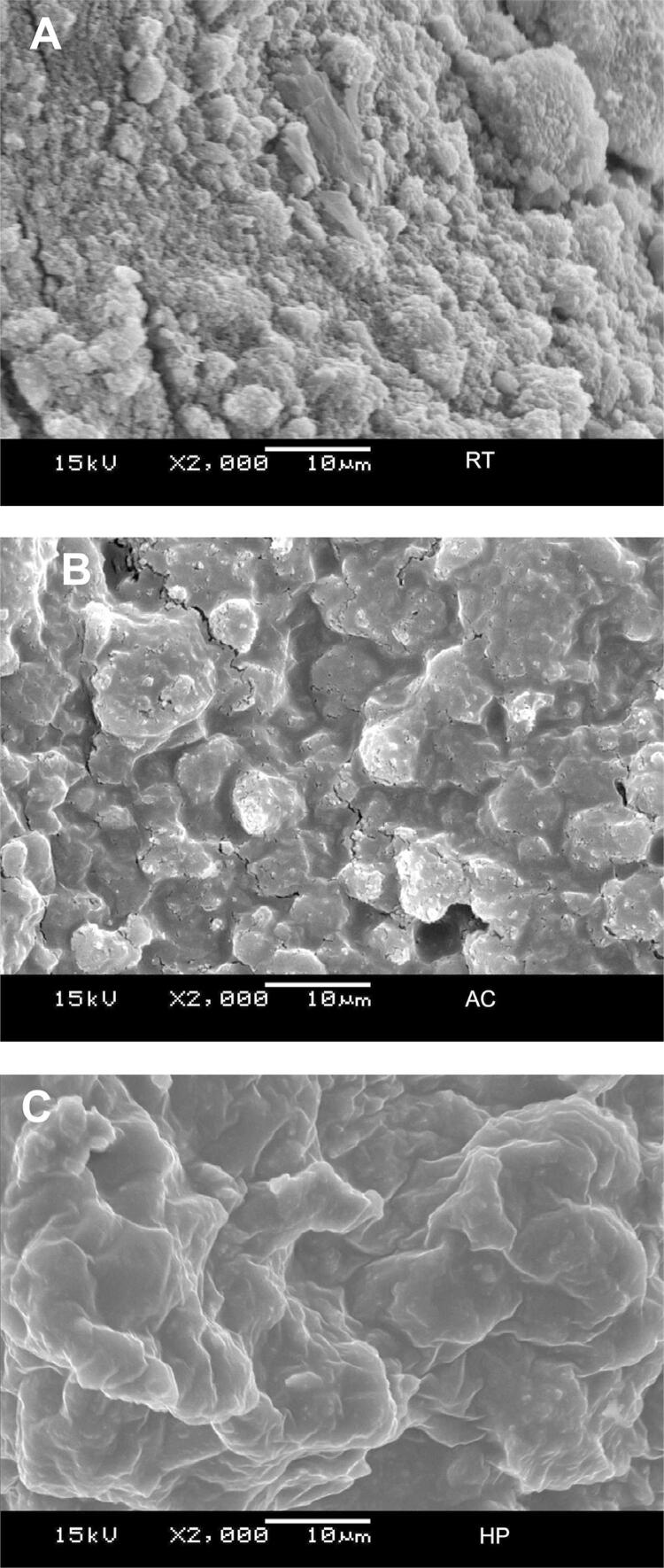
Images obtained by scanning electron microscopy at 2000× magnification. (A) RT: Regular toothpaste; (B) AC: Activated Charcoal Toothpaste; (C) HP: Hydrogen Peroxide Toothpaste

Table 1 shows the analysis of the physical characteristics of the tested toothpastes (pH, analysis of the weight% of solid particles, and RDA). The images of the filler particle characterization are shown in Figure 3. RT (Figure 3A) showed irregularly shaped particles and nanoparticles. AC (Figure 3B) presented more regular spherical particles surrounded by a matrix, and HP (Figure 3C) revealed larger particles than those of the other toothpastes.

#### Roughness

**Figure 4:**
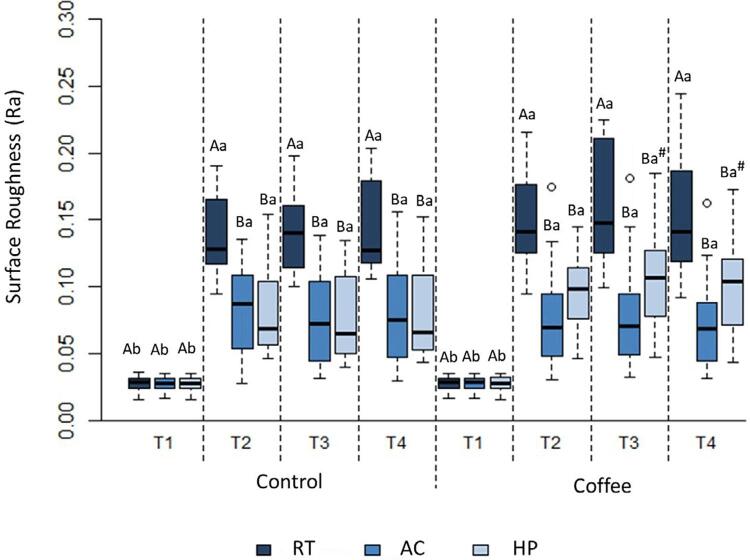
Median (minimum and maximum) values of roughness according to toothpaste, staining and time

RS: Remineralizing solution; RT: Regular toothpaste; AC: Activated Charcoal Toothpaste; HP: Hydrogen Peroxide Toothpaste. T1: before brushing; T2: after brushing; T3: after staining or after 7 days in AS; T4: after staining or after 14 days in RS. Distinct letters (uppercase letters in rows and lowercase letters in columns) differ among one another (p≤0.05). The symbol (#) indicates differences among non-staining groups of the same toothpaste and time periods (p≤0.05).

Figure 4 shows the median roughness value. At T1, no significant difference was observed between the groups regarding roughness values (p>0.05). At T2, the Ra values were higher for RT than AC and HP (p<0.05). The Ra values did not change significantly at T3 or T4 (p>0.05). The HP/Coffee sample showed greater roughness than HP at T3 and T4 (p<0.05).

#### Color

Table 2 shows the mean color differences (ΔE_ab_ and ΔE_00_). At T2-T1, the color difference was significantly higher for the RT than the HP group (p<0.05), with no difference between RT and AC (p<0.05). At T3-T1, the ΔE_ab_ and ΔE_00_ values were significantly higher in the staining groups, with no significant difference in the toothpastes (p>0.05) between the non-staining and staining groups (p>0.05). At T4-T1, the ΔE_ab_ and ΔE_00_ values were significantly higher in the staining groups (p<0.05), with no significant difference between the three toothpastes regarding color variation at this period (p>0.05).

**Table 2:** Median (minimum and maximum) value of ΔEab and ΔE00 according to toothpaste, exposure to remineralizing solution or coffee, and time

	Time			Toothpaste		p-value
			**RT**	**AC**	**HP**	
	T2-T1	RS	0.61(0.08;1.43)^Aa^	0.55(0.09; 1.25)^Aba^	0.37(0.05;1.11)^Ba^	0.0158
		Coffee	1.04(0.55; 2.16)^Aa^	0.62(0.32;1.25)^Aa^	0.34(0.06;0.83)^Ba^	<0.0001
ΔE_a.b_	p-value		0.053	0.3438	0.7251	
	T3-T1	RS	0.89(0.30;1.39)^Ab^	0.90(0.21;1.64)^Ab^	0.63(0.16;2.23)^Ab^	0.1725
		Coffee	3.46(2.16;4.65)^Aa^	2.77(1.43;4.31)^Aa^	3.43(1.85;5.71)^Aa^	0.0336
	p-value		<0.0001	<0.0001	<0.0001	
	T4-T1	RS	1.36(0.52;2.88)^Ab^	1.65(1.04;2.42)^Ab^	1.39(0.34;2.86)^Ab^	0.0557
		Coffee	2.84(2.00;3.72)^Aa^	2.71(1.86;4.21)^Aa^	2.57(1.41;4.23)^Aa^	0.1605
	p-value		<0.0001	<0.0001	<0.0001	
	T2-T1	RS	0.57(0.10;1.24)^Aa^	0.55(0.08;1.07)^ABa^	0.34(0.05;0.91)^Ba^	0.0302
		Coffee	0.88(0.49;1.84)^Aa^	0.65(0.31;1.06)^Aa^	0.37(0.07;0.71)^Ba^	<0.0001
ΔE_00_	p-value		0.0087	0.2448	0.7251	
	T3-T1	RS	0.77(0.39;1.30)^Ab^	0.89(0.26;1.49)^Ab^	0.64(0.13;1.75)^Ab^	0.1653
		Coffee	2.96(1.86;3.86)^Aa^	2.44(1.45;3.65)^Aa^	2.90(1.75;4.82)^Aa^	0.0536
	p-value		<0.0001	<0.0001	<0.0001	
	T4-T1	RS	1.17(0.44;2.41)^Bb^	1.58(0.89;2.17)^Ab^	1.21(0.33;2.31)^Bb^	0.0062
		Coffee	2.51(1.89;3.17)^Aa^	2.34 (1.82;3.72)^Aa^	2.29 (1.35;3.84)^Aa^	0.2739
	p-value		<0.0001	<0.0001	<0.0001	

RS: Remineralizing solution; RT: Regular Toothpaste; AC: Activated Charcoal Toothpaste; HP: Hydrogen Peroxide Toothpaste.

T1: before brushing; T2: after brushing; T3: after staining or after seven days in AS; T4: After staining or after 14 days in RS.

#### Gloss unit (GU)

**Figure 5:**
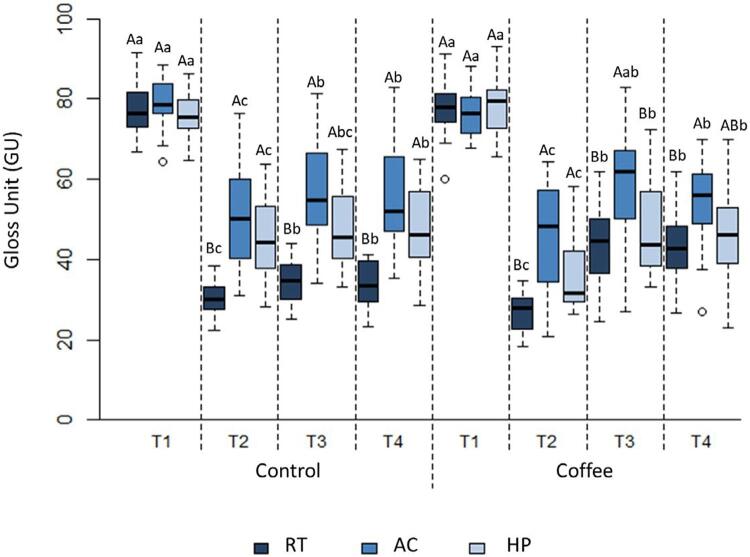
Median (minimum and maximum) values of gloss according to toothpaste, staining, and time

RS: Remineralizing solution; RT: Regular Toothpaste; AC: Activated Charcoal Toothpaste; HP: Hydrogen Peroxide Toothpaste. T1: before brushing; T2: after brushing; T3: after staining or after seven days in AS; T4: After staining or after 14 days in RS. Distinct letters (uppercase letters in rows and lowercase letters in columns) differ among one another (p≤0.05). The symbol (#) indicates differences among non-staining groups of the same toothpaste and time periods (p≤0.05).

Figure 5 shows the median gloss value. At T1, no significant difference was observed among the groups (p>0.05). At T2, the gloss value decreased significantly among the three toothpastes (p<0.05), in which the decrease was significantly higher in the AC and HP than the RT groups (p<0.05). At T3, the gloss values increased significantly and remained unchanged at T4 (p<0.05). The gloss value was significantly higher in the AC than the HP and RT groups (p<0.05). Moreover, a significant increase was observed in the gloss values at T4 compared to T2 (p<0.05).

The gloss values for RT at T3 and T4 were significantly higher in the coffee stain groups than in the control groups (p<0.05). Conversely, the gloss value for HP at T2 was significantly lower in the coffee stain group than in the control group (p<0.05).

## Correlation Coefficient

Table 3 shows the relationship between the GU logarithm and the average surface roughness. A negative correlation was found between gloss and roughness (p<0.05), except for RT/Coffee at T3 and T4.

**Table 3:** Relation between the logarithm of the surface gloss and the average surface roughness

Staining	Toothpaste	Time	Correlation coefficient	p-value
Control	RT	T1	-0.76	0.0001
		T2	-0.72	0.0004
		T3	-0.49	0.0288
		T4	-0.73	0.0002
	AC	T1	-0.49	0.0276
		T2	-0.84	<0.0001
		T3	-0.73	0.0002
		T4	-0.83	<0.0001
	HP	T1	-0.78	<0.0001
		T2	-0.76	0.0001
		T3	-0.66	0.0016
		T4	-0.77	<0.0001

Coffee	RT	T1	-0.76	0.0001
		T2	-0.54	0.0128
		T3	-0.37	0.1092
		T4	-0.44	0.0503
	AC	T1	-0.52	0.0199
		T2	-0.88	<0.0001
		T3	-0.84	<0.0001
		T4	-0.86	<0.0001
	HP	T1	-0.66	0.0015
		T2	-0.77	<0.0001
		T3	-0.75	0.0001
		T4	-0.80	<0.0001

RT: Regular toothpaste; AC: Activated Charcoal Toothpaste; HP: Hydrogen Peroxide Toothpaste; RS: Remineralizing solution. T1: before brushing; T2: after brushing; T3: after staining or after seven days in RS; T4: after staining or after 14 days in RS.

## Scanning electron microscope (SEM)

**Figure 6:**
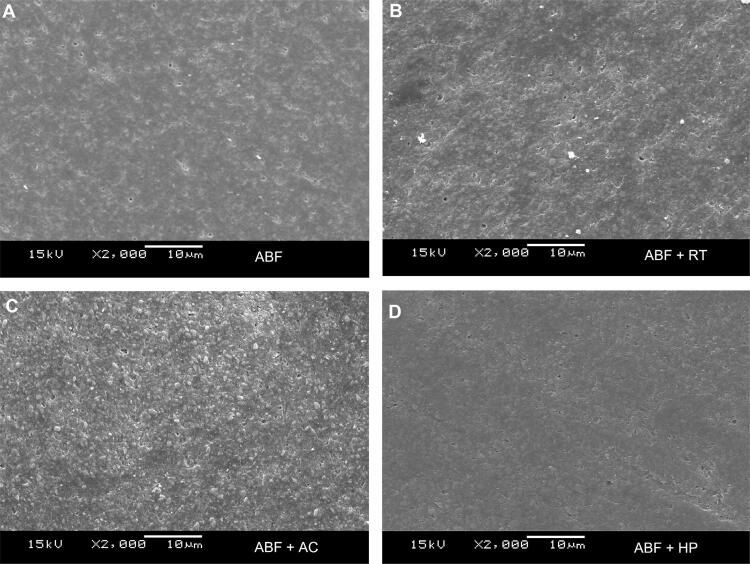
Images obtained by scanning electron microscopy at 2000× magnification of bulk-fill composite resin not exposed to toothpaste and after six months of simulated toothbrushing. (A) Untreated bulk-fill resin; (B) exposure to regular toothpaste; (C) exposure to active charcoal toothpaste; (D) exposure to hydrogen toothpaste

The SEM analysis presented in Figure 6 showed morphological characteristics and alterations of the bulk-fill composite resin (Aura Bulk Fill - ABF) after its exposure to the toothpastes (RT, AC and HP). Figure 3A presents the untreated surface of ABF. A qualitative analysis of these images shows a smoother and less polished surface with more irregular clusters of inorganic particles dispersed in the matrix. After 5,000 cycles of simulated toothbrushing (Figures 6B, 6C, 6D), the surface integrity was compromised, with micromorphological changes in the composite surface and greater exposure of filler particles for all the toothpastes. Figure 6B shows the composite surface after brushing with a regular toothpaste. The image shows a greater exposure of filler particles on the composite resin surface after brushing than before brushing (Figure 6A). The exposure to activated charcoal toothpaste (Figure 6C) resulted in lower irregularities on the resin composite surface (more homogeneous distribution of inorganic particles dispersed in the matrix) compared to Figure 3B. Figure 6D shows a polished composite surface after brushing with hydrogen peroxide toothpaste.

## Discussion

Whitening toothpastes have become popular with both patients and manufacturers.^29^ Our study evaluated the possible effect of an increased roughness and extrinsic pigmentation of a bulk-fill composite resin after using whitening toothpastes. The first null hypothesis was accepted: the whitening toothpastes did not cause greater roughness than regular toothpaste. The second null hypothesis was partially accepted: although the whitening toothpastes did not promote a color alteration greater than regular toothpaste, and all toothpastes decreased the composite gloss, the whitening toothpastes showed a higher composite gloss than regular toothpaste.

Whitening toothpastes are commonly speculated as more abrasive than conventional pastes since they contain more abrasive ingredients.^2^ This investigation evaluated the effect of three toothpastes containing different active ingredients (Table 1): RT (regular toothpaste), HP (hydrogen peroxide-based toothpaste); and AC (carbon-based toothpaste) on the surface of ABF composite.

In this study, a tooth brushing machine was used to simulate the *in vivo* environment and proved to be very effective in evaluating the efficacy of different abrasives used in toothpaste formulations, as shown in other studies.^4,11,12,25^ Individual brushing patterns range widely; hence, it is difficult to establish a relationship between the number of brushing cycles and the equivalent amount of brushing time.^4^ A previous clinical study^26^ found that a person’s brushing average ranges from 25–30 cycles per day, equivalent to 4562–5475 cycles in six months. Based on previous studies associating whitening toothpastes and composite resin,^30-31^5,000 brushing cycles were performed in this study, or an equivalent of six months of toothpaste use.

This study found a significant increase in the roughness values for all the toothpastes after brushing compared to baseline (Figure 4). This shows that even a regular toothpaste could change an important property of the composite; thus, the first null hypothesis was rejected. Toothbrushing could degrade a composite surface by a three-body wear process, by removing the polymer matrix layer (the smoothest) to expose the filler particles.^12^ It can also increase the abrasion effect, since toothbrush bristles do not wear the surface of the material as uniformly as the discs or polishing pads used in tooth finishing and polishing procedures.^12^

Composite degradation is material-dependent and based on the resin matrix and the type and size of the filler particle.^12^ The composite resin analyzed in the study features Bis-GMA and TEDGMA monomers (Table 1); composites with these monomers tend to have lower hardness.^12^ Initially, a qualitative SEM analysis of the ABF composite surface (Figure 6A) showed a smooth, less polished surface with irregular clusters of inorganic particles dispersed in the matrix. After simulated brushing of the ABF surface (Figure 6 B-D), its integrity was found to be compromised, and the micromorphological changes in the composite surface resulted in greater exposure of filler particles. Toothbrushing with toothpaste accelerates the degradation of composites by exposing the filler particles, hence compromising the polish and increasing the roughness.^12^

Regarding the clinical significance of the SEM used in this study, it was evident that the surface roughness of the analyzed composite resin changed according to the type of toothpaste used, pointing out that the roughness was significantly higher for the RT than the AC and HP groups (Figure 4). The abrasiveness created by the toothpaste during brushing is influenced by parameters such as type of brush, load applied during brushing, and physical characteristics of the abrasive particles, such as pH, type, shape, size, distribution particle hardness,^3^ and weight% of the solid particles,^3,32-33^ as supported by the results discussed in this study.

This study used a standard soft-bristled toothbrush, the type most recommended by clinicians.^32,33^ The load was set at 200 g, which corresponds to several reference figures, including the mid-range of the minimum clinically recommended amount determined in clinical trials, the maximum amount recommended by ISO for *in vitro* tests, and the amount corroborated by previous studies.^25,32^ Thus, the results were explored based on the physical characteristics (pH, characterization of particle and weight% of solid particles) of the formulations analyzed herein.

The pH can interfere with the degradation of the polymeric matrix layer of the composite resin.^34^ However, all the slurry solutions prepared in this study had a pH above 7 (RT=7.65; AC=7.12; HP=8.25); hence, this factor cannot be associated with changes in the composite surface or the composite resin properties.

All the toothpastes analyzed in this study contained silica or hydrated silica in their composition (Table 1), combined with other abrasives, and possibility with different ingredient concentrations not described on the toothpaste packaging. Silica-based toothpaste has excellent cleaning ability, hence stain removal ability.^2,25^ However, silica ranges in size and hardness, thus limiting the comparison to only those types of abrasives present in the formulations,^2^ as indicated by the SEM images. Nevertheless, different toothpastes with the same abrasive ingredients do not necessarily have the same abrasiveness,^35^ as corroborated by the results present in this study.

A previous study^2^ reinforced the concept that abrasiveness is not limited to the type of abrasive ingredient or to the degree of association between the abrasives, but mainly to the physical characteristics of the mineral, such as particle size and shape, as well as other variables that may be present in the oral cavity. For example, when silica is formed by fine particles with regular shapes, it preserves its characteristic of a light abrasive mineral, but when it consists of coarse and irregular particles, it is highly abrasive.^36^

SEM images (Figure 3) showed that the tested toothpastes contained particles ranging in size and shape and included or excluded agglomerates. RT showed a higher concentration of particles and microparticles with a pointed irregular shape (Figure 3A). This feature can be correlated with previous studies reporting that irregular particles produced rough surfaces.^12^ In contrast, AC (Figure 3B) and HP (Figure 3C) toothpastes showed more regular spherical particle clusters surrounded by a matrix.

This study evaluated the percentage weight of solid particles (%) of the tested toothpastes. This solid content consists primarily of abrasives, which are responsible for cleaning or polishing tooth structures.^37^ The percentage weight of solid particles ranged among the groups, thus indicating that the toothpastes had different compositions. RT presented a greater content of solids (57.94%), followed by HP (54%) and AC (42%). The solid residues may determine the potential to alter the surface, since the higher values were a sign of changes in Ra.^37^

A previous study showed that brushing with AC toothpastes could increase the roughness of composite resins,^38^ promoting morphological changes as seen in the SEM image (Figure 6C). These toothpastes have been gaining popularity among patients, especially for all the aesthetic appeal involved, and it is believed that activated charcoal added to toothpaste binds to stains and deposits on tooth surface, which would then be more easily removed by brushing.^39^

HP contains 2% hydrogen peroxide. Hydrogen peroxide produces high-energy free radicals that may have an adverse effect on the resin-filler-particle interface and cause detachment of the filler-matrix particle,^38^ thus aggravating crack propagation and significantly increasing the Ra, although in our study, this alteration was not greater than that promoted by the RT. The SEM analysis of this sample showed loss of sharpness of the organic matrix and detachment of the filler particles (Figure 6D).

The literature has reported that abrasives also have a negative effect on the color of restorations.^31^ In this regard, color change is caused by an increase in the porosity of the restoration surface.^31^ A quality control approach for visual and instrumental findings in dentistry and in standardizing research is essential to determine if a difference in color can be perceived, and if this difference is acceptable.^5^ Perceptibility/acceptability thresholds were considered for ΔE_ab_ and ΔE_00_ (1.2/2.7 and 0.8/1.8, respectively), in order to evaluate overall color change.^5-23^The ΔE_ab_ and ΔE_00_ values after brushing were significantly higher for the RT than the HP group. The value of ΔE_ab_ was below the limit of 1.2, which is considered imperceptible. However, the ΔE_00_ value was slightly above the perceptibility threshold (0.88), thus making this color change more visually perceptive than ΔE_ab._^23^

Possibly, the organic matrix of ABF was more easily removed because it was brushed with a toothpaste that had a higher percentage weight of solid particles (RT), which caused more light to be scattered, and the color change to be more greatly perceived. Although the color of AC is black, the presence of other components (Table 1), such as water, in the charcoal-based toothpaste (AC) may have diluted the activated charcoal and reduced the color change, making this toothpaste show not much higher ΔE_ab_ and ΔE_00_ values than other toothpastes.

The gloss parameter, associated with color, deeply influences the survival rates of restorations, since this parameter is influenced by how light is reflected on the surface.^40^ In our study, the composite gloss decreased significantly for all the toothpastes after brushing, and RT had lower gloss than AC and HP (Figure 5). In this regard, the decrease in surface gloss could be related to roughness, but the strength of this relationship varies depending on the value of the roughness.^40^ A statistical analysis of the correlation coefficient between gloss and roughness was performed in this study (Table 3) and revealed a significant negative correlation between gloss and roughness. Thus, an increase in roughness was correlated with a decrease in gloss, a finding that does not corroborate a previous study.^24^The rougher the material, the greater the amount of light scattered on its surface, thus leading to a decrease in gloss.^40^

Coffee was used as a staining agent in this study, since it is a beverage consumed daily and has a strong potential to stain restorative materials.^27-28^ After the staining protocol was applied to the composite, no statistical difference was found in its roughness post-brushing against pre-brushing (Figure 4). However, a difference was found between the group exposed to both HP and coffee and the group exposed only to HP (not to coffee). Coffee contains over 22 acids, including citric and acetic acids, as confirmed by the low pH of the solution analyzed in this study (pH=4.9). This difference could be attributed to the interaction of these acids with the hydrogen peroxide contained in HP.

Exposure to coffee resulted in higher ΔE_ab_ and ΔE_00_ values compared to the non-staining group (Table 2). The results showed ΔE_ab_ and ΔE_00_ values above the limit of 2.7 and 1.8, indicating that coffee immersion caused clinically perceptible color changes. Moreover, we found no statistical difference between the toothpastes with respect to overall color change. The presence of compounds like water, alcohol, and other solvents in coffee may promote the adsorption of pigments present in coffee, derived from the polymer-solvent affinity that induces staining.

After the sample was immersed in coffee (T3), the gloss (Figure 4) was significantly higher than it was immediately after brushing (T2), and it remained unchanged seven days after staining. This result probably occurred because coffee particles filled the irregularities^26^ formed by brushing, thus making the surface a little smoother and improving light reflection and increasing the gloss values.

The gloss value was significantly higher in the AC group than in the RT and HP groups. After seven days, the gloss was higher for BC than RT. Variations based on the decrease in gloss occur when a fraction of the light hitting the composite resin is not fully transmitted, but lost to factors such as intrinsic absorption, pores, and roughness, thus affecting the optical property.^40^ Considering that RT had higher initial roughness values, it can be speculated that this change may have further hampered gloss recovery. Any study model on resin color stability should unquestionably consider the effects of the chemical structure of the materials, as well as the composition of the toothpaste.

This study had some limitations, other conditions that can increase surface roughness and loss of gloss were not answered. Acidic staining, brushing force, and amount of toothpaste used by each patient are variations that can be replicated in clinical studies. This study did not test the effect of different bristle types; we considered one brushing force and a model that considers only a coffee exposure protocol. This way, studies focusing on different toothbrush bristles (hard, soft, and extra soft), several brushing forces, and pigmentation protocols should be conducted.

## Conclusion

Within the limitations of this study, it could be concluded that:

Whitening toothpastes promoted an increased roughness but did not increase it more than a regular toothpaste.

All toothpastes promoted a decreased gloss, and coffee exposure promoted higher color alteration in the bulk-fill composite regardless of the type of toothpaste previously used.
